# Biological and clinical impact of membrane EGFR expression in a subgroup of OC patients from the phase IV ovarian cancer MITO-16A/MANGO-OV2A trial

**DOI:** 10.1186/s13046-023-02651-y

**Published:** 2023-04-11

**Authors:** Luca Forlani, Loris De Cecco, Vittorio Simeon, Biagio Paolini, Marina Bagnoli, Sabrina Chiara Cecere, Anna Spina, Eleonora Citeroni, Eliana Bignotti, Domenica Lorusso, Laura Arenare, Daniela Russo, Carmine De Angelis, Laura Ardighieri, Giosuè Scognamiglio, Michele Del Sesto, Germana Tognon, Daniela Califano, Clorinda Schettino, Paolo Chiodini, Francesco Perrone, Delia Mezzanzanica, Sandro Pignata, Antonella Tomassetti

**Affiliations:** 1 Integrated Biology of Rare Tumors, Department of Experimental Oncology, Fondazione IRCCS Istituto Nazionale Dei Tumori, Milan, Italy; 2 Department of Mental Health and Public Medicine, Section of Statistics, Università Degli Studi Della Campania Luigi Vanvitelli, 80138 Naples, Italy; 3 Department of Pathology, Fondazione IRCCS Istituto Nazionale Dei Tumori, Milan, Italy; 4 Urogynaecological Medical Oncology, Istituto Nazionale Tumori IRCCS, Fondazione G. Pascale, 80131 Naples, Italy; 5 Microenvironment Molecular Targets Unit, Istituto Nazionale Tumori IRCCS, Fondazione G. Pascale, 80131 Naples, Italy; 6 Division of Obstetrics and Gynecology, ASST Spedali Civili Di Brescia, Brescia, Italy; 7 Angelo Nocivelli Institute of Molecular Medicine, ASST Spedali Civili of Brescia- University of Brescia, Brescia, Italy; 8 Department of Life Science and Public Health, Catholic University of Sacred Heart Largo Agostino Gemelli, and Fondazione Policlinico Universitario A. Gemelli, IRCCS, Rome, Italy; 9 Clinical Trials Unit, Istituto Nazionale Tumori IRCCS, Fondazione G. Pascale, 80131 Naples, Italy; 10 Department of Clinical Medicine and Surgery, Federico II University, Via Sergio Pansini 5, 80131 Naples, Italy; 11 Department of Pathology, Azienda Socio Sanitaria Territoriale Spedali Civili Di Brescia, Brescia, Italy; 12 Pathology Unit, Istituto Nazionale Tumori IRCCS, Fondazione G. Pascale, 80131 Naples, Italy

**Keywords:** Ovarian cancer, Bevacizumab, EGFR, Immunohistochemistry, Microarray, Bioinformatics

## Abstract

**Background:**

Validated prognostic biomarkers for anti-angiogenic therapy using the anti-VEGF antibody Bevacizumab in ovarian cancer (OC) patients are still an unmet clinical need. The EGFR can contribute to cancer-associated biological mechanisms in OC cells including angiogenesis, but its targeting gave disappointing results with less than 10% of OC patients treated with anti-EGFR compounds showing a positive response, likely due to a non adequate selection and stratification of EGFR-expressing OC patients.

**Methods:**

EGFR membrane expression was evaluated by immunohistochemistry in a cohort of 310 OC patients from the MITO-16A/MANGO-OV2A trial, designed to identify prognostic biomarkers of survival in patients treated with first line standard chemotherapy plus bevacizumab. Statistical analyses assessed the association between EGFR and clinical prognostic factors and survival outcomes. A single sample Gene Set Enrichment-like and Ingenuity Pathway Analyses were applied to the gene expression profile of 195 OC samples from the same cohort. In an OC in vitro model, biological experiments were performed to assess specific EGFR activation.

**Results:**

Based on EGFR-membrane expression, three OC subgroups of patients were identified being the subgroup with strong and homogeneous EGFR membrane localization, indicative of possible EGFR out/in signalling activation, an independent negative prognostic factor for overall survival of patients treated with an anti-angiogenic agent. This OC subgroup resulted statistically enriched of tumors of histotypes different than high grade serous lacking angiogenic molecular characteristics. At molecular level, among the EGFR-related molecular traits identified to be activated only in this patients’ subgroup the crosstalk between EGFR with other RTKs also emerged. In vitro, we also showed a functional cross-talk between EGFR and AXL RTK; upon AXL silencing, the cells resulted more sensitive to EGFR targeting with erlotinib.

**Conclusions:**

Strong and homogeneous cell membrane localization of EGFR, associated with specific transcriptional traits, can be considered a prognostic biomarker in OC patients and could be useful for a better OC patients’ stratification and the identification of alternative therapeutic target/s in a personalized therapeutic approach.

**Supplementary Information:**

The online version contains supplementary material available at 10.1186/s13046-023-02651-y.

## Background

Ovarian cancer (OC) is a very aggressive disease diagnosed in advanced stage in 80% of the cases, with the median survival being less than 5 years. The OC management is now evolving from an approach which includes surgery followed by platinum (pt)-based therapy to the selection of patients likely to benefit from different therapeutic modalities currently based on PARP inhibitors (PARPi), for pt-sensitive patients, or on anti-angiogenic agents as Bevacizumab (BEVA) [1]. The efficacy and safety of adding BEVA to standard front-line treatment of ovarian cancer were demonstrated in two randomized phase III trials, the GOG-0218 [2] and the ICON7 [3]. Adding BEVA to carboplatin and paclitaxel chemotherapy significantly improved progression-free survival (primary endpoint) in both trials, although no significant impact on overall survival was detectable in the overall population of either trial. Nevertheless, subgroup analysis from both GOG-218 and ICON7 clinical trials showed an OS benefit in a subgroup of patients classified as ‘high risk’ (defined by suboptimally debulked stage III-IV disease) [4, 5]. The results of these two studies led to the approval by the European Commission of BEVA in combination with standard chemotherapy (carboplatin and paclitaxel) as front-line treatment for women with advanced stages ovarian cancer. Although the introduction of PARPi in first-line maintenance has changed the therapeutic management and, consequently, the outcome of OC patients, the identification of prognostic/predictive biomarkers guiding the use of BEVA remains an important clinical need. The phase IV MITO16a-MaNGO-OV2 single arm clinical trial was therefore designed with a translational primary endpoint aimed at the evaluation of potential molecular prognostic factors helping in the selection of patients who could best benefit from BEVA treatment [6–9].

Among the OC molecular subtypes which have been associated to a higher risk of relapse, ‘mesenchymal’ and ‘proliferative’, are characterized by an angiogenic-like signature [10] and resulted those having the best benefit from BEVA [11]. At the protein level, co-expression of VEGFR2 and other receptor tyrosin kinases (RTKs) such as EGFR and MET, expressed on endothelial and tumor cells, respectively, has been associated to aberrant VEGF-A expression and intrinsic resistance to BEVA [12]. Targeting with anti-EGFR drugs decreased VEGF expression through a down-regulation of VEGF promoter thus negatively affecting angiogenesis [13]. Conversely, an association between EGFR mutations and resistance to BEVA was identified in clear cell renal carcinomas and OC patients [14]. Very recently, targeting both EGFR and VEGFR1 by a bispecific decoy receptor, able to capture both EGF-like and VEGF ligands, showed great anti-tumor activity in a preclinical in vivo model of lung carcinoma [15]. These data indicate a clear link between EGFR activation, VEGF/VEGFR1 axis and angiogenesis, in tumors different then OC.

In OC patients, EGFR overexpression has been also associated with a lack of response to chemotherapy [16, 17]. Although EGFR is considered to be a key therapeutic target in many types of cancers [18], for reasons that are still unclear, targeting EGFR in OC patients resulted in a very poor response not correlated to EGFR expression [19–22]. EGFR has been found expressed in an estimated 10–70% of OCs and, although its expression was originally associated with advanced stage disease and poor prognosis [23], more recently this association to clinical parameters or survival was not confirmed [24]. The controversial results obtained in studies assessing the clinical impact of EGFR expression may possibly be due to a general evaluation of its expression without a precise attention to its cellular localization (cytoplasm vs. membrane) that can be linked to different functions, with the EGFR membrane expression able to activate out/in signalling leading to growth and invasion [25]. In a small cohort of high grade serous OC (HGSOC) patients (*n* = 23) we found distinct EGFR sub-cellular localizations with 26% of samples with a clear cytoplasmic localization and 22% of samples with strong membrane expression [26] thus raising the hypothesis that different cellular localizations could impact on EGFR’s biological functions. Accordingly, we identified a ligand-dependent activation of membrane-associated EGFR leading to IL-6, IL-8 and PAI-1 production [26], cytokines contributing to inflammation but also to angiogenesis [27].

To assess in OCs whether membrane localization of EGFR, indicative of possible EGFR out/in signalling activation, could impact on the prognosis of OC patients treated with standard front line chemotherapy and an anti-angiogenic agent we took advantage of samples from OC patients treated with BEVA and collected for translational purposes from the phase IV MITO16a-MaNGO-OV2 trial. Since for 63% of the samples analyzed by immunohistochemistry for EGFR expression gene expression profile was also available, we performed a bioinformatics analysis for assessing the molecular networks associated to EGFR membrane expression.

## Methods

### Patients’ cohort

This study is part of the translational analysis on the OC biopsies of the MITO16A/MaNGO-OV2 clinical trial, coordinated by the Clinical Trials Unit at Istituto Nazionale Tumori IRCCS “Fondazione G. Pascale” of Naples. MITO16A is a multicenter, phase IV, single arm trial of BEVA in combination with carboplatin and paclitaxel, designed to searching for prognostic biomarkers as one of its primary endpoints [6]. The study was conducted in accordance with the ethical standards and according to the Declaration of Helsinki and National and International guidelines. The study was approved by ethical committees at each participating center.

### Immunohistochemistry (IHC)

Tissue macroarrays (TMAs) were prepared as previously described [9]. Briefly, formalin-fixed paraffine-embedded FFPE blocks were collected from 385 out of the 398 enrolled patients. The primary tumor was the preferential site requested for translational analyses, but, when this was not available, blocks from synchronous peritoneal secondary localizations were used. Three cores from each patient were enclosed in the TMAs, thus addressing both the issue of tumor heterogeneity and the risk of sample loss. Four μm-thick TMA sections were cut, sent to the recipient lab and processed for EGFR staining. Slides were deparaffinized in xylol and serially rehydrated, and after antigen retrieval with Proteinase K for 20 min, the samples were stained with mouse monoclonal anti-human EGFR antibody (Clone E30, Dako, Denmark) diluited 1:25. Immunoreactions were visualised using streptavidin–biotin-peroxidase (Thermo Fisher Scientific) and the DAB Chromogen System (Dako Agilent Technologies) and counterstained with Carazzi haematoxylin. Two observers (BP, pathologist, and AT, experienced in IHC) independently evaluated the sections and scored each TMA core. Both were blinded to the clinicopathological parameters and clinical outcomes of the patients. Discrepant scores between the two observers, reported in less than 5% of cases, were discussed to achieve a consensus. The final score was obtained taking into account only the intensity of the membrane EGFR staining, suggestive of receptor activation: no staining, negative (defined as M0); focal membrane staining in ≤ 30% of tumor cells (defined as M); 100% membrane staining of the tumor cells in all replicates present in the TMA (defined as MM). Within the negative samples, those stained slightly and diffusely in the cytoplasm were considered M0 (*n* = 24). Images were acquiring with the Aperio Image Scope software (Leica Biosystems, Nussloch, Germany) at a magnification corresponding to 20 × objective. Images were processed using Adobe Photoshop software.

IHC for Ki-67 was performed with ready to use antibody clone 30–9 (Ventana Medical Systems Inc) on Bond Max Automatic Immunostainer (Leica Biosystems, GmbH, Wetzlar, Germany). The evaluation of immunostaining was performed separately by two independent observers (LA, gynecopathologist and EB, biologist experienced in IHC) and results were expressed as a percentage of positive tumor cells in the overall neoplastic population. In case of discordant results, slides were reviewed together using a dual head microscope and a consensus was agreed upon.

### Gene-expression

After pathological revision, starting from FFPE tissue blocks, cores were selected to represent at least a percentage > 70% of tumor cells, no significant signs of necrosis and to avoid excessive presence of stromal tissue. RNA from 195 patients of MITO16A/MaNGO-OV2 was isolated using Qiagen RNeasy FFPE kit (Qiagen, Hilden, Germany) and quality checks were performed as previously described [7].

Gene expression profiles were performed using the GeneChip WT Pico kit (Affymetrix, Thermo Fisher Scientific) following the guidelines for FFPE material. Probes were hybridized on human Clariom D chips for 16 h at 45 °C; after washing and staining, the chips were scanned with an Affymetrix Gene Chip Scanner 3000 7G. The Affymetrix Clariom D chips were designed to detect genes, exons, and alternative splicing events from > 540,000 transcripts. Primary data were acquired using the Affymetrix GeneChip Command Scan Control version 4.0 (Thermo Fisher Scientific, Waltham, Massachusetts, MA, USA). The generated CEL files were analyzed for an additional quality check using Affymetrix Expression Console Software (version 1.4), which normalized array signals using Signal Space Transformation (SST) and a robust multiarray averaging (RMA) algorithm and summarized data at gene level. The final data matrix includes about 28,000 unique genes.

Further analyses were performed using R [(R Foundation for Statistical Computing, Vienna, Austria) http://www.R-project.org.], version 4.1.2, to challenge in our dataset the angiogenic-related signature developed by Bentink et al. [28].

### Gene set enrichment analysis (GSEA) and Ingenuity pathway Analysis (IPA)

Single sample enrichment scores were carried out for the 195 patients of MITO16A/MaNGO-OV2 cohort with both EGFR evaluation and gene expression profile available.

The scores were obtained applying the Gene Set Variation Analysis (GSVA) method, a non-parametric, unsupervised approach to assess gene set enrichment, available within the *gsva* function of the open source Bioconductor GSVA package (version 1.44.2) for R^27^. The complete list of the gene sets, the included genes, and their annotations is available in Supplementary Tables 5 and 4. The lists of genes for each gene set were downloaded from the GSEA database via the R package *msigdbr* that was generated from Molecular Signatures Database MSigDB v7.5.1 (released January 2022). Five gene sets were related to EGFR processing and 16 gene sets to EGFR signalling. The latest gene sets were selected among those present in the collection ‘Gene Ontology’, all contained EGFR gene and not more than 120 genes. The complete list of the gene sets, the included genes, and their annotations is available in Supplementary Table 4, Table 3 and Supplementary Table 5. Scores from the MITO16A/MaNGO-OV2 cohort were then compared across the groups defined by EGFR membrane expression. Statistical significance of the differences in enrichment among the EGFR subgroups was assessed applying the Student’s T-test to the GSVA scores and a *P*-value < 0.05 was considered significant. IPA (Ingenuity Systems, 2021 release) (QIAGEN Inc., Hilden, Germany), a software leveraging a manually reviewed repository of biological interactions and functional annotations was used to analyze the signalling pathways, cellular location, function and network connections of the identified genes. For each gene set, the genes included in the signature were considered and the Log Fold Changes obtained comparing the MM subgroup vs (M0 + M) subgroup were considered as input values for the IPA analysis.

### Cell lines and reagents

The EOC cell lines used in this study were: SKOV3 and OV-90 and TOV-112D from ATCC; OVCAR5 and OVCAR4, provided by Dr. Camalier (NCI-NIH, USA); OAW42, kindly provided by Dr Ulrich (Dr. A Ullrich, Martinsried, Germany); PEO6, provided by Dr. López-Guerrero (Valencia, Spain). Cells were maintained in RPMI 1640 medium or EMEM (for OAW42) (Sigma Aldrich, St. Louis, MO) supplemented with 10% FCS (Hyclone, Logan, UT), 1% L-glutamine, at 37 °C in a humidified atmosphere of 5% CO2. Cells were genotyped at Eurofins Genomic Europe (Ebersberg, Germany) and were routinely confirmed to be mycoplasma-free by a MycoAlert Mycoplasma Detection Kit (Lonza, Basel, Switzerland). Human recombinant EGF was from PeproTech (London UK); human recombinant Gas6 from R&D systems, Inc. (Minneapolis, MN, USA). EGF (20 ng/ml) and/or GAS6 (500 ng/ml) stimulation at indicated time points was performed in serum-free medium on 24 h starved cells.

### Confocal immunofluorescence

Cells grown adherent on glass slide coverslips, were fixed with 2% paraformaldehyde for 20 min and permeabilized for 10 min in PBS containing 0.1% Tween 20. The immunoreactions were performed as described [29]. The primary antibodies were: mouse MINT-5 [30] for EGFR; rabbit anti-AXL (C89E7, Cell Signaling, 1:100). Confocal microscopy was carried out using a Leica TCS SP8 X confocal laser scanning microscope (Leica Microsystems GmbH, Mannheim, Germany). Images were acquired in the scan format 512 × 512 pixels in a single plane using a HC PL APO CS2 63X/1.30 oil-immersion objective and a pinhole always set to 1 Airy unit and analyzed using Leica LAS AF rel. 3.3 (Leica Microsystems GmbH) software. Images were processed using ImageJ and Adobe Photoshop software.

### Western blotting

Western blotting was performed as already described [29]. Lysates were separated on a 3–8% SDS-PAGE. The primary antibodies used were: mouse anti-phosphorylated EGFR (Tyrosine 1068) (1H12, Cell Signaling. 1:500); rabbit anti-EGFR (#2232, Cell Signaling,1:500); rabbit anti-phosphorylated AXL (Tyrosine 703) (D12B2, Cell Signaling, 1:300); rabbit anti-AXL (C89E7, Cell Signaling, 1:1000); mouse anti-β-actina (clone C4, sc-47778. Santa Cruz Biotechnology).

### Erlotinib treatment on AXL silenced cells

Cells were transfected with 40 nmol/ml small-interfering RNA (siRNA) duplex specific for AXL [catalogue n. s1847 (siRNA #1); s1845 (siRNA #2), ThermoFisher Scientific, Waltham, MA, USA] or control siRNA (Quiagen-Xeragon, Germantown, MD). 1 × 10^5^ cells were seeded in a 24-well plate and transfected with 40 pmol/ml siRNA using Lipofectamine 2000 (Invitrogen, ThermoFisher Scientific) according to the manufacturer’s protocol. After 24 h, cells were collected and seeded in 96-well plate (5 × 10^3^ cells). Upon adhesion, cells were treated with erlotinib at different doses (0, 1.25, 2.50, 5, 10, 20 μM) for 48 h. Cell viability was measured using CellTiter-Glo® Luminescent Cell Viability Assay (Promega, Madison, WI).

### Statistical analysis

Continuous variables were described with median values and interquartile range, qualitative variables were expressed in terms of absolute numbers and relative frequency. The associations between biomarker and the clinical prognostic factors were investigated using the chi-squared test or the Fisher's exact test as indicated.

The prognostic effect of EGFR staining was evaluated using progression free survival (PFS) and overall survival (OS) as endpoints. PFS was defined as the time elapsing from the inclusion into the study to the first occurrence of either death for any cause or disease progression. OS was defined as the time elapsing from the inclusion into the study and death for any cause. Kaplan–Meier curves were drawn for PFS and OS and compared with a two-sided log-rank test.

Furthermore, Cox proportional models were performed reporting hazard ratios (HRs) and 95% confidence intervals (CIs).

After a first univariable analysis, the biomarker was analysed in a multivariable model using as covariates: age (as category < 65 vs. ≥ 65), ECOG performance status (PS) (0 vs. 1–2), residual disease (None; ≤ 1 cm; > 1 cm; not operated), FIGO stage (III vs. IV) and tumor histology (high grade serous vs. other). Covariates were chosen according to the model defined in the manuscript reporting the clinical results of this trial [6]. In each model EGFR biomarker was analysed by setting M0 as a reference. Data were analyzed using R software version 4.1.2.

GraphPad Prism software (GraphPad Software, San Diego, CA) was used to analyze all in vitro data. Differences between mean values were determined by ANOVA. Each experiment was performed at least three times for each condition; representative experiments are shown.

## Results

### Evaluation of EGFR membrane expression in the OC cohort

**Fig. 1:**
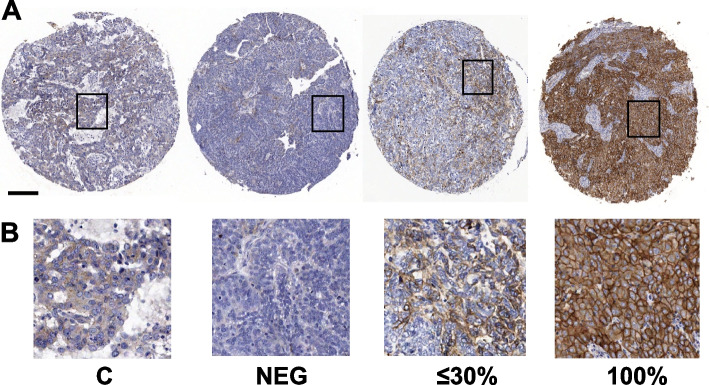
Representative images of OC sections stained with the anti-EGFR antibody. C, cytoplasmic staining; NEG, no staining. Percentage of the clear cell membrane staining is reported below pictures. **A** Representative images of an entire section within the TMA is reported. Black empty box, area shown in panel B. Bar, 200 μm. **B** Higher magnification of each section in panel A

Immunohistochemical evaluation of EGFR membrane expression was performed on 310/398 patients’ samples representing 78% of the patients enrolled in the trial (Supplementaty Fig. 1). The cohort analyzed was comparable to the overall study population with a slightly lower rate of patients not operated at baseline (Supplementary Table 1). Considering the biological relevance of EGFR out/in signalling in tumors, we therefore focused our attention on EGFR membrane staining on the entire cohort and on the HGSOC group. Forty-nine percent of all OCs was negative for EGFR membrane staining (hereafter defined as M0); 46,8% showed a focal membrane EGFR staining (M) in no more than 30% of the section analyzed while 4,2% had a strong and homogeneous EGFR staining (MM), (Table 1 and Fig. 1 for representative images of EGFR staining). EGFR staining was significantly associated (Fisher’s exact test *p* = 0.001) with tumor histotype; interestingly, EGFR MM stained only 6/265 HGSOC cases compared to 7/45 non-HGSOC cases. When the same analyses were performed categorizing patients for EGFR negative and positive staining, without differentiating for percentage of membrane expression, no significant associations were seen with clinical-pathological characteristics and prognosis (Supplementary Tables 2 and 3). EGFR activation can lead to increased proliferation [25], however no significant association was observed between EGFR membrane staining and nuclear expression of the mitotic marker Ki67, included into the list of biomarkers analyzed in the MITO16a-MaNGO-OV2 translational study (Supplementary Fig. 2).

**Table 1:** Association of membrane EGFR localization with the clinical prognostic characteristics of the OC cohort

	**EGFR**			
	*M0* *N (%)*	*M* *N (%)*	*MM* *N (%)*	*p-value*
	(*N* = 152)	(*N* = 145)	(*N* = 13)	
**Age**				
< 65	105 (69.1%)	104 (71.7%)	11 (84.6%)	0.522
> = 65	47 (30.9%)	41 (28.3%)	2 (15.4%)	
**Stadio (3 vs 4)**				
III	119 (78.3%)	120 (82.8%)	11 (84.6%)	0.611
IV	33 (21.7%)	25 (17.2%)	2 (15.4%)	
**ECOG**				
0	120 (78.9%)	117 (80.7%)	11 (84.6%)	0.938
1–2	32 (21.1%)	28 (19.3%)	2 (15.4%)	
**Tumor histology**				
HGSOC	136 (89.5%)	123 (84.8%)	6 (46.2%)	** < 0.001**
Other	16 (10.5%)	22 (15.2%)	7 (53.8%)	
**Residual**				
None	66 (43.4%)	58 (40.0%)	7 (53.8%)	0.787
< = 1 cm	27 (17.8%)	33 (22.8%)	3 (23.1%)	
> 1 cm	49 (32.2%)	45 (31.0%)	2 (15.4%)	
No surgery	10 (6.6%)	9 (6.2%)	1 (7.7%)

### Clinical impact of membrane EGFR expression

**Fig. 2:**
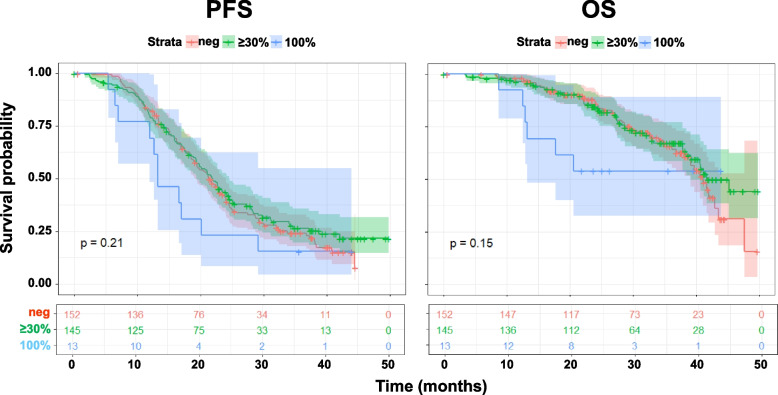
Kaplan-Meyer analysis evaluating the progression free survival (PFS) and the overall survival (OS) of patients from the MITO16a-MaNGO-OV2i stratified according to EGFR membrane expression by immunohistochemical staining

Kaplan–Meier curves of PFS and OS of MITO16A/MANGO-OV2A patients stratified for EGFR membrane expression, clearly showed that MM patients experienced a shorter median PFS time (13.3 months) and median OS time (not achieved) as compared to other two groups (M0 and M) which showed an almost super imposable median time of PFS and OS (Fig. 2). Kaplan-Meyer curves showed that MM subgroup, when compared to M0 subgroup, had a trend to worse prognosis although not statistically significant in univariable analysis due to a small number of patients constituting this subgroup [PFS, HR 1.56 (95% CI 0.84–2.90) *p* = 0.16; OS, HR 1.99, (95% CI 0.85–4,65) *p* = 0,11]. In the multivariable analysis MM membrane EGFR expression, compared to M0 subgroup, resulted to be an independent prognostic factor for OS [HR = 2.84 (95% C.I. 1.18–6.38) *p* = 0.019] but not for PFS [HR = 1.79 (95% C.I. 0.95–3.36) *p* = 0.07] (Table 2).

**Table 2:** Multivariable survival analysis for the immunohistochemical membrane EGFR staining

	**PFS**			**OS**		
	**HR**	**(95% CI)**	**P** value	**HR**	**(95% CI)**	***P*-value**^a^
**EGFR**						
M0	1			1		
M	0.9	(0.69–1.19)	0.47	0.92	(0.61–1.38)	0.68
MM	1.79	(0.95–3.36)	0.07	2.84	(1.18–6.83)	**0.019**
**Age**						
< 65y	1			1		
≥ 65y	0.89	(0.65–1.21)	0.45	0.79	(0.51–1.24)	0.31
**ECOG PS**						
0	1			1		
1–2	1.47	(1.05–2.06)	0.02	1.91	(1.19–3.06)	**0.0075**
**FIGO stage**						
IIIB-IIIC	1			1		
IV	2.17	(1.57–3)	< 0.0001	1.81	(1.14–2.88)	**0.011**
**Residual disease**						
None	1			1		
≤ 1 cm	1.47	(1.02–2.1)	**0.037**	1.61	(0.92–2.84)	0.09
> 1 cm	1.87	(1.34–2.63)	**0.0003**	2.14	(1.3–3.55)	**0.003**
No surgery	3.23	(1.88–5.52)	** < 0.0001**	2.97	(1.41–6.26)	**0.0043**
**Tumor histology**						
HGSOC	1			1		
Other	1.28	(0.87–1.88)	0.21	1.17	(0.67–2.03)	0.59

^a^In bold are significant *p* value from Cox multivariable analysis

### Transcriptional characterization of MM OC subgroup: single sample enrichment approach

Based on the levels of EGFR membrane expression, we hypothesized that the receptor might trigger different molecular pathways of activation. To identify membrane EGFR-driven molecular pathways, we took advantage of the gene expression profile from 195 cases of the 310 OCs evaluated for immunohistochemical staining and having similar clinical-pathological characteristics (Supplementary Table 1). Among the 195 samples, 5 showed an MM EGFR receptor staining pathway with a subgroup distribution for hystotype comparable to that observed in the 310 samples analyzed for IHC (Supplementary Fig. 3A).

At first, we evaluate whether the three EGFR-related OC subgroups displayed the angiogenesis-related molecular signature already described for OC [28]. All of the MM OC samples belonged to the *Non Angiogenic* subtype while among the M0 and M EGFR-expressing samples about 20% were classified as *Angiogenic* (Supplementary Fig. 3B).

**Fig. 3:**
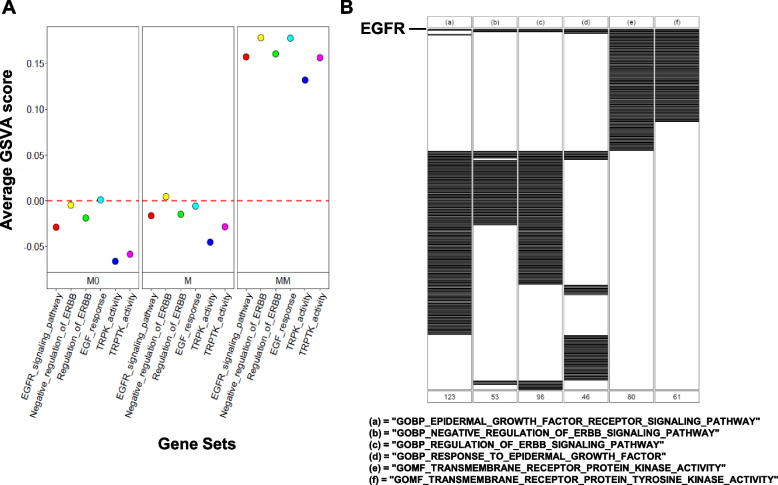
**A** Graphical representation of the mean GSVA scores for the EGFR-related gene sets (see Table 3 for GSVA scores and GSEA). The red line highlights the 0 score. Different dots’ colors represent different gene sets, as reported. For the corresponding GSEA nomenclature refers to Table 4. **B** Graphical representation of the overlap among the gene sets significantly enriched in MM staining subgroup defined in the MITO16a-MaNGO-OV2 trial. The three gene sets (a), (d) and (e) were selected for further analysis since they include all the EGFR-related genes. Each black line represents a gene; for each gene set the number of genes is reported at the bottom. The names of the gene sets are reported below the scheme

To further investigate on the molecular portraits of OC patients stratified according to EGFR-expression, we used an approach based on GSVA which is a single sample GSEA-based method more suitable in the case of classes constituted by few samples. We selected 21 gene sets among those relevant for EGFR processing (*n* = 5) and signalling (*n* = 16; see Materials and Methods for gene set selection). We first applied GSVA using the 5 gene sets related to EGFR processing (Supplementary Table 4, first 5 gene sets): none of these gene sets showed statistically significant differences in enrichment among the three OC subgroups. When we applied GSVA to the gene sets containing EGFR, 10 gave no statistically significant results (Supplementary Table 4); six of them resulted significantly enriched only in MM subgroup (Table 3 and Fig. 3A). No statistically significant differences in enrichment were observed applying Student’s t test to the comparison between the other two EGFR-generated OC subgroups.

**Table 3:** Single-sample GSVA analysis on EGFR-containing gene sets

**Gene Set**	**EGFR subgroups**^a^		**Mean GSVA score**		***P*-value**^b^
**(*nomenclature reported in ***Fig. 3A**)**	**A**	**B**^**c**^	**A**	**B**	
GOBP_EPIDERMAL_GROWTH_FACTOR_RECEPTOR_SIGNALING_PATHWAY (*EGFR_signaling_pathway*) (113) {123]^d^	0	M	-0,0288	-0,0162	0,588
	**0**	**MM**	**-0,0288**	**0,1576**	**0,007**
	**M**	**MM**	**-0,0162**	**0,1576**	**0,009**
GOBP_NEGATIVE_REGULATION_OF_ERBB_SIGNALING_PATHWAY (*Negative_regulation_of_ERBB*) (47) [53]	0	M	-0.0049	0.0042	0.783
	**0**	**MM**	**-0.0049**	**0.1789**	**0.024**
	**M**	**MM**	**0.0042**	**0.1789**	**0.028**
GOBP_REGULATION_OF_ERBB_SIGNALING_PATHWAY (*Regulation_of_ERBB*) (87) [96]	0	M	-0,0195	-0,0151	0,857
	**0**	**MM**	**-0,0195**	**0,1605**	**0,0003**
	**M**	**MM**	**-0,0151**	**0,1605**	**0,0002**
GOBP_RESPONSE_TO_EPIDERMAL_GROWTH_FACTOR (*EGF_response*) (44) [46]	0	M	0,0001	-0,0067	0,84
	**0**	**MM**	**0,0001**	**0,1768**	**0,038**
	**M**	**MM**	**-0,0067**	**0,1768**	**0,033**
GOMF_TRANSMEMBRANE_RECEPTOR_PROTEIN_KINASE_ACTIVITY (*TRPK_activity*) (79) [80]	0	M	-0,059	-0,0287	0,178
	**0**	**MM**	**-0,059**	**0,1569**	**0,018**
	**M**	**MM**	**-0,0287**	**0,1569**	**0,03**
GOMF_TRANSMEMBRANE_RECEPTOR_PROTEIN_TYROSINE_KINASE_ACTIVITY (*TRPTK_activity*) (60) [61]	0	M	-0,0667	-0,0456	0,348
	**0**	**MM**	**-0,0667**	**0,1309**	**0,032**
	**M**	**MM**	**-0,0456**	**0,1309**	**0,046**

^a^EGFR-expressing: M0, EGFR negative; M, 30% EGFR membrane expression; MM, 100% EGFR expression

^b^In bold significant different p values by Student’s t test

^c^A and B indicate the group comparison and the relative GSVA scores

^d^()﻿number of genes present in the MITO16A dataset; [] number of genes in the genesets

**Table 4:** Upstream regulators, included in the EGFR-related gene sets, predicted by IPA. The complete lists of their target genes are reported in Sup Table 6

Upstream Regulator	Molecule Type	Activation z-score	*P*-value of overlap
***EGF_Response***^***a***^			
AGT	growth factor	2,535	1.97E-03
ERK	group	2,378	5.11E-07
TNF	cytokine	2,298	3.45E-04
OSM	cytokine	2,236	7.57E-04
AR	ligand-dependent nuclear receptor	2,222	6.52E-05
IGF1	growth factor	2,199	1.54E-08
IL4	cytokine	2,177	2.48E-03
nicotine	chemical drug	2,136	8.64E-05
TP63	transcription regulator	2,122	1.68E-09
EGFR	kinase	2,122	4.51E-07
HDAC2	transcription regulator	2,000	2.72E-04
***EGFR_Singnaling_Pathway***^***a***^			
Insulin	group	3,415	4.69E-05
8-bromo-cAMP	chemical reagent	2,547	2.87E-04
GNA12	enzyme	2,414	1.33E-07
medroxyprogesterone acetate	chemical drug	2,361	3.41E-02
AGT	growth factor	2,201	1.45E-07
TCF7L2	transcription regulator	2,200	1.61E-02
NFE2L2	transcription regulator	2,179	8.76E-02
MYC	transcription regulator	2,095	5.07E-02
estrogen	chemical drug	2,025	1.59E-05
PTP4A1	phosphatase	2,000	2.95E-03
***TRPK***^***a***^			
FSH	complex	3,102	1.47E-08
MYC	transcription regulator	2,689	2.54E-05
FGF2	growth factor	2,639	1.99E-10
PDGF BB	complex	2,560	2.03E-04
8-bromo-cAMP	chemical reagent	2,534	2.76E-08
tretinoin	chemical—endogenous mammalian	2,495	1.66E-09
EGF	growth factor	2,370	3.32E-07
EPAS1	transcription regulator	2,364	2.83E-05
KLF4	transcription regulator	2,271	1.40E-09
sphingosine-1-phosphate	chemical—endogenous mammalian	2,236	1.69E-04
NANOG	transcription regulator	2,219	2.49E-05
topotecan	chemical drug	2,219	5.05E-04
bleomycin	biologic drug	2,219	8.12E-04
Z-LLL-CHO	chemical—protease inhibitor	2,219	8.13E-03
HGF	growth factor	2,211	7.79E-03
ESR1	ligand-dependent nuclear receptor	2,027	1.80E-09
TNF	cytokine	2,010	7.88E-09

^a^The detailed nomenclature of the EGFR-related gene sets is reported in Table 3

To better characterize at molecular level the OC patients with MM EGFR expression and experiencing the worst prognosis following BEVA treatment, based on the gene overlap among the six EGFR-related gene sets (Fig. 3B and Supplementary Table 5 for the full list of genes), we decided to further analyze EPIDERMAL_GROWTH_FACTOR_RECEPTOR_SIGNALING_PATHWAY, RESPONSE_TO_EPIDERMAL_GROWTH_FACTOR and TRANSMEMBRANE_RECEPTOR_PROTEIN_KINASE_ACTIVITY gene sets (identified respectively as *a*, *d* and *e* in Fig. 3B).

### EGFR pathway is activated in the MM OC subgroup: analysis by IPA

**Fig. 4:**
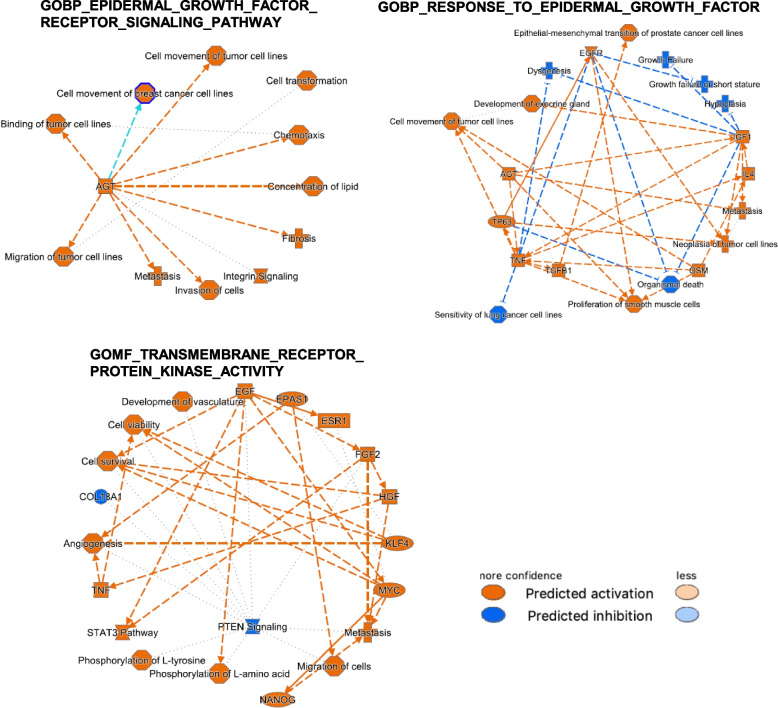
Graphical representation of the genes/functions predicted by IPA performed using the differential gene expression derived by comparing MM vs M and MO groups of patients. IPA was run using the log fold changes of the genes up- or down-modulated in MM vs the other two subgroups and included in the EGFR-related gene sets reported in Table 4. See Methods for details of the analysis. The name of each gene set is reported above each scheme. Panel in the right side, prediction legends

To give further insight in the transcriptional traits characterizing the MM OC subgroup, we performed an IPA analysis using the list of differential gene expression derived by comparing MM vs M and MO groups of patients. Upon IPA, basically the functions reported in the summaries for datasets *a* and *d* were cell movement, invasion and cell transformation, and the epithelial-mesenchymal transition (EMT) (Fig. 4) all related to cancer. For the EPIDERMAL_GROWTH_FACTOR_RECEPTOR_SIGNALING_PATHWAY gene set, AGT (encoding for angiotensinogen) was the key molecule. AGT is an essential component of the renin-angiotensin (RAS) system [31], a potent regulator of blood pressure, body fluid and electrolyte homeostasis (Table 4). For RESPONSE_TO_EPIDERMAL_GROWTH_FACTOR gene set, which includes all EGFR ligand (EGF, EREG, HGEGF, TGFA), AGT emerged as key molecule together with TP63, TNF and TGFB1. A different view is given by the analysis of the third gene set, TRANSMEMBRANE_RECEPTOR_PROTEIN_KINASE_ACTIVITY, which only contains genes encoding the RTKs: besides the activation of the cancer-related functions and EGF, other upstream regulator (FGF2 and HGF, and EPAS1, ESR, KLF4 and MYC, growth and transcription factors, respectively) were predicted to be activated in the MM OC subgroup (Table 4). Specifically, for the upstream regulators likely involved in regulation of the EGFR-related genes, Supplementary Table 6 reports in details the predicted activated genes for each gene set with activation Z-score ˃2 and a *p* value ≤ 0.01. Among all and besides the activators already mentioned above, there is GNA12, encoding for G-alpha subunits of heterotrimeric GTP-binding proteins (G proteins) that play important roles in human physiology. For the third gene set, to be noted the gene for the stemness transcription factor NANOG, and PDGF BB, a gene related to angiogenesis, encoding a member of the protein family comprised of both platelet-derived growth factors (PDGF) and VEGF.

**Fig. 5:**
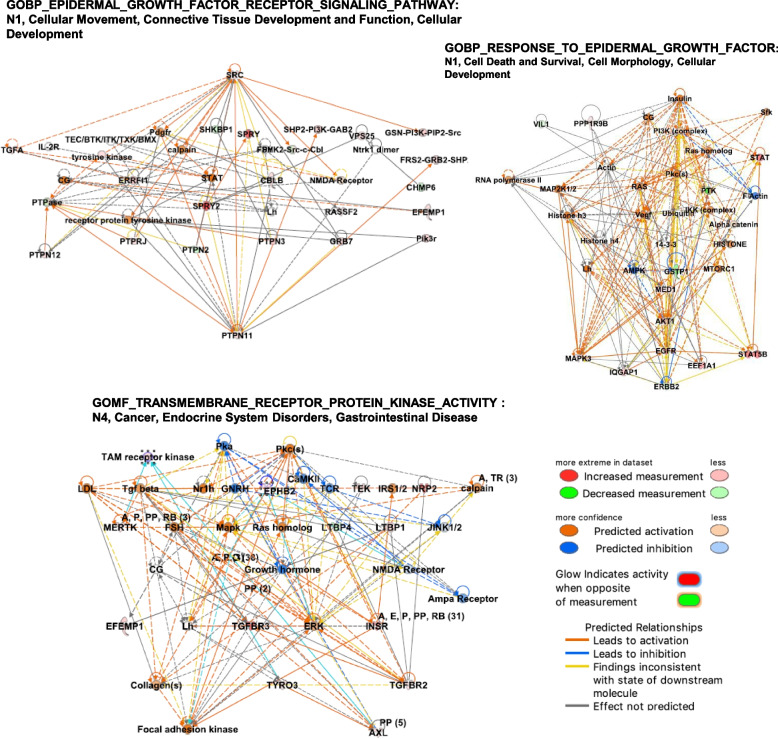
Among the networks obtained by IPA and listed in Supplementary Table 7, representative significant networks are reported. The name of each gene set and of the selected network is reported above each scheme. Panel in the right side, prediction legends

The network analysis shows the interactions between molecules in each of the selected dataset (Table 5 for predicted networks, and Supplementary Table 7 for the list of the genes in these networks). The hierarchical representation of the highest ranked networks for the first two gene sets showed SRC and AKT as key activated genes, as expected for EGFR canonical pathway activation (Fig. 5). In addition, the prediction of the downstream effector STAT5A, whose gene is also upmodulated, is suggestive of possible activation of inflammation that we have already demonstrated in vitro and in OC patients [26, 32]. For the last selected gene set the associated predicted networks were mainly associated to cancer (Supplementary Table 7) and in one of those emerged the TAM family of RTKs (TYRO3, AXL, MER) and in particular a slight AXL up-modulation in the gene expression (Fig. 5). Indeed, we also contributed to show AXL expression associated to OC aggressiveness and the presence of AXL ligand, GAS6, in the OC microenvironment [33].

**Table 5:** Networks generated by IPA for the EGFR-related gene sets. The list of gene included in each network is reported in Sup. Table 7

Networks	Score	Focus Molecules	Top Diseases and Functions
***EGF_Response***^***a***^			
1	31	16	Cellular Movement, Connective Tissue Development and Function, Cellular Development
2	31	16	Hair and Skin Development and Function, Organ Development, Protein Degradation
3	28	15	Cellular Assembly and Organization, Cellular Function and Maintenance, Infectious Diseases
4	21	12	Endocrine System Disorders, Organismal Injury and Abnormalities, Reproductive System Disease
5	19	11	Cellular Movement, Nervous System Development and Function, Cellular Development
***EGFR_Signaling_Pathway***^***a***^			
1	24	11	Cell Death and Survival, Cell Morphology, Cellular Development
2	21	10	Cellular Development, Embryonic Development, Organismal Development
3	16	8	Cancer, Developmental Disorder, Embryonic Development
4	9	5	Infectious Diseases, Hematological Disease, Cardiovascular Disease
5	9	5	Connective Tissue Disorders, Dermatological Diseases and Conditions, Developmental Disorder
***TRPK***^***a***^			
1	42	19	Post-Translational Modification, Cancer, Organismal Injury and Abnormalities
2	31	15	Cancer, Organismal Injury and Abnormalities, Respiratory Disease
3	28	14	Post-Translational Modification, Cell Signaling, Developmental Disorder
4	23	12	Cancer, Endocrine System Disorders, Gastrointestinal Disease
5	16	9	Cell Morphology, Embryonic Development, Hair and Skin Development and Function

^a^The detailed nomenclature of the EGFR-related geene sets is reported in Table 3

**Fig. 6:**
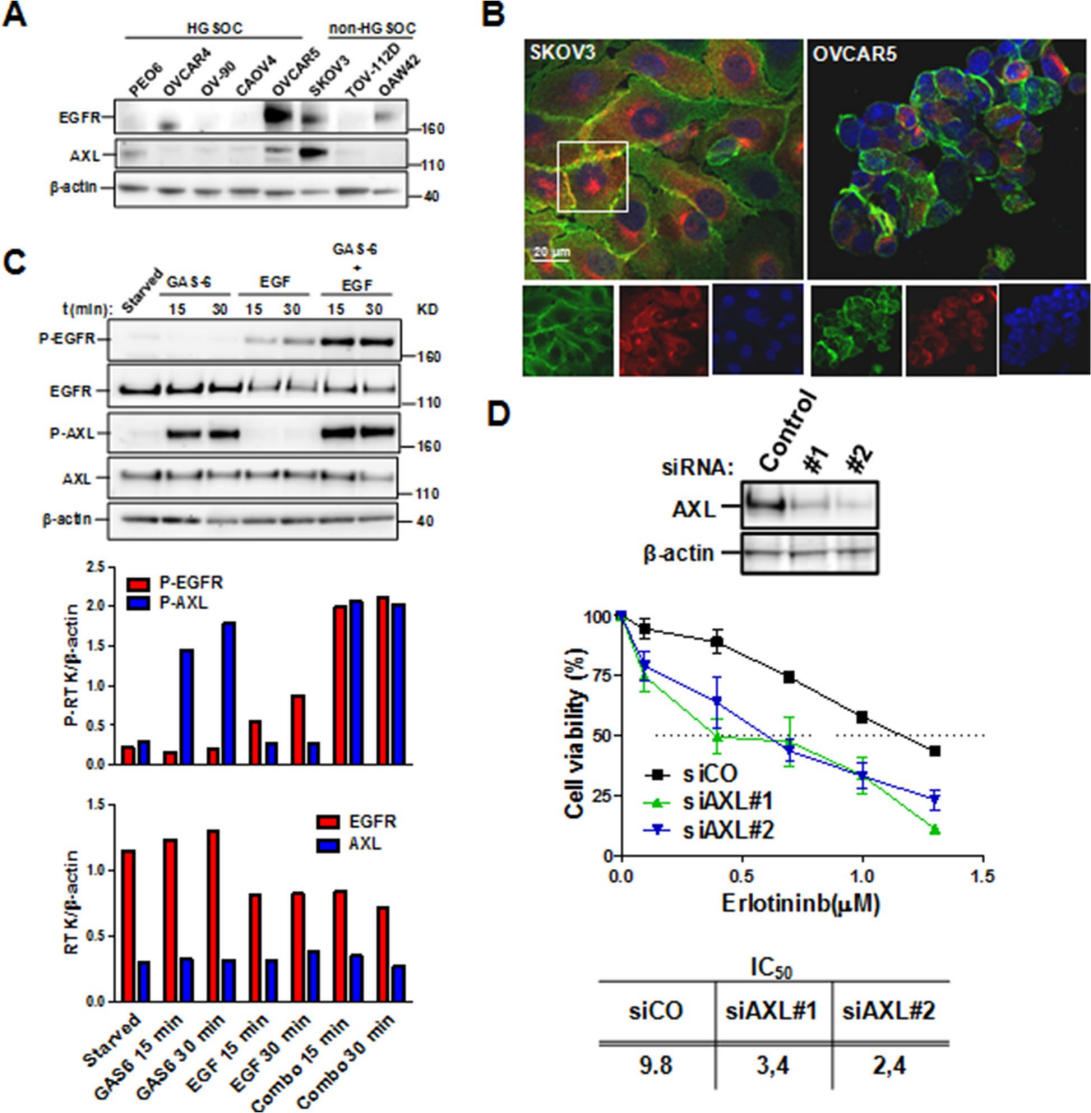
**A** Western blotting for EGFR and AXL expressions in lysates from OC cell lines representative of HGSOCs or non-HGSOCs cell lines [34]. SKOV3 and OVCAR5 cells co-expressed both EGFR and AXL. **B** Confocal immunofluorescence showing EGFR and Axl expressions; only SKOV3 showed both EGFR (green) and AXL (red) expressions on the cell membrane with several regions of co-localization (white box indicates one of those regions). In OVCAR5 cells only EGFR is clearly expressed on the membrane. SKOV3 was chosen for further analysis. Upper, merge staining; lower. single staining, Nuclei were stained with DAPI. **C** upper. Western blotting of lysated from SKOV3 cells stimulated with GAS6 or EGF alone or in combination; lower, densitometric analysis for phoshorylated (P-EGFR and P–AXL) or total RTKs (EGFR and AXL). As expected, the total amount of EGFR decreased upon ligand stimulation [35]. No changes in the amount of AXL were detected upon GAS6 stimulation. **D** Erlotinib susceptibility of AXL silenced SKOV3 cells. Upper, western blotting showing the amount of silenced AXL in SKOV3 lysates upon transfection with two different siRNAs (#1 and #2). Lower, viability of SKOV3 cells treated with control siRNA (siCO) or with specific Axl siRNAs (siAxl#1 and #2) and then treated with erlotinib at different concentration. Statistical evaluation by ANOVA, *p* ≤ 0.001. Refer to Methods section for detailed procedure. The table below reports the IC50 values of siRNA transfected cells. The experiments were performed at least three times

Hence, to assess a possible functional interaction between EGFR and another RTK, we here explored a possible cross-talk between EGFR and AXL. OVCAR5 and SKOV3 cells were identified as the cell lines expressing both receptors (Fig. 6A) but only SKOV3 cells showed both EGFR and AXL expressed on the cell membrane with several regions of co-localization (Panel B). In OVCAR5 cells only EGFR was clearly expressed on the membrane. Accordingly to the latest data, SKOV3 cells stimulation with EGF and GAS6 combined treatment induced a 2-fold higher EGFR phosphorylation respect to the treatment with EGF alone, while GAS6 alone only caused AXL phosphorylation (Panel C). As shown in Panel D, upon silencing of AXL with two different siRNAs, SKOV3 cells were more sensitive to the anti-EGFR drug erlotinib with great decrease of the IC_50_ (Panel D) indicating a functional relationship between these two RTKs. Altogether, these data suggested a possible targeting of EGFR together with AXL in OC cells expressing both functional RTKs on the cell membrane.

## Discussion

Our study assessed the relevance of EGFR membrane expression, rather than global expression, on the prognosis of OC patients using the case material derived from the MITO-16A/MaNGO-OV2A trial designed with translational end points for advanced OC patients treated with front-line chemotherapy combined with BEVA. Based only on EGFR-membrane expression, three OC subgroups of patients were identified being the MM staining (high and homogeneous EGFR membrane localization) an independent negative prognostic factor for OS. Furthermore, analyzing the gene expression profile of 195/310 patients’ samples stained for EGFR, in the MM subgroup specific EGFR-related networks resulted activated with prediction of new possible upstream activators of EGFR-related genes as well as new crosstalk with RTKs. As proof-of-principle, we also showed in an in vitro model a functional cross-talk between EGFR and AXL suggesting a possible targeting on both RTKs.

The purpose of the translational study proposed for MITO-16A/MaNGO-OV2A trial was to explore the potential role of clinical and biologic factors in identifying those patients who benefit most, in terms of PFS and OS, from carboplatin-placlitaxel plus BEVA schedule in first line setting, followed by BEVA maintenance [6]. EGFR membrane expression on tumor cells was among the biomarkers analyzed on the TMAs prepared from patients’ biopsies since several evidences previously demonstrated that EGFR activation can induce VEGF expression within the tumor microenvironment thus increasing OC angiogenesis [36]. Here, we showed that the MM EGFR membrane expressing subgroup of OC patients with the worst outcome did not display an angiogenic-related portrait, observation that might, at least in part, biologically explain why these patients less benefit from BEVA.

Based on the assumption that out/in activated signalling starts from the receptor expressed on the membrane, the adding value of our study is the detailed evaluation of EGFR expression in one of the largest OC cohort so far analyzed for this biomarker. Immunohistochemical analysis of EGFR expression gave previously unclear results in OCs likely depending on the antibodies used and on interpretation of staining often considering the comprehensive cytoplasmic and membrane EGFR expressions. Another study, analyzing samples from nearly 500 OC patients, did not succeed in finding an association between cytoplasmic or membrane EGFR localization and the clinical characteristics as well as impact on patients’ survival [24]. However, in this report a different EGFR classification was adopted, basically considering negative vs. positive EGFR staining and therefore preventing a comparison with our classification. Indeed, in our cohort we clearly defined EGFR membrane expression and found that about 50% of the OCs express EGFR on tumor cell membrane and 4,2% of them showed high and homogeneous EGFR membrane expression. Although the number of samples was low, these patients resulted to be those with the worst OS outcome. We can hypothesize that in these patients the tumor cells are strongly dependent from EGFR signalling activation. Analysis of the association between EGFR membrane expression and clinical parameters showed that only MM subgroup was significantly associated with non-HGSOC histological types, which represent the minority of the OC patient population. These data might also explain the low percentage of OC patients experiencing a benefit from anti-EGFR therapies [19–21]. We can also speculate that the exploitation of anti-EGFR therapies might be restricted to these OC patients, especially non-HGSOC subtypes. Although in the present cohort non-HGSOC group included a very heterogenous group of tumors arising from different type of cells and having different molecular and phenotypic characteristics as well as susceptibility to standard chemotherapy [37], they might anyway have in common high EGFR membrane expression on the membrane leading to specific network activation as those identified by these analyses.

We are aware that the limitation of this study is that the MM subgroup of OC represents only a low percentage of OC patients present in our cohort and unfortunately a validation on an independent cohort is not suitable at the moment. On the other hand, we like to give emphasis to the fact that the patients with MM EGFR expression present not only a significant bad outcome when treated with chemotherapy and BEVA, but they also experienced a lower response rate. Indeed, in the patients eligible for RECIST assessment we observed a trend (not statistically significant) showing a gradual decrease in the percentage of responders (from 79 to 56%) and a gradual increase in the percentage of non responders (from 21 to 44%) from M0 to MM EGFR expression. The effort of searching for a possible, even retrospective, validation cohort of patients will be surely pursued. Despite the above mentioned limitation, our data become interesting in the view of a personalized treatment approach, prompting us to assess the peculiar molecular characteristics for the MM OC subgroup. Our GSVA analysis on the gene expression profile, available for 63% of the EGFR analyzed population, identified three EGFR-related gene sets strictly reminiscent of EGFR signalling activation in this OC subgroup. Despite the controversies on the role of EGFR as prognostic marker and therapeutic target, these data showed that in an OC subgroup EGFR can impinge several different biological mechanisms such as growth, migration/invasion and induction of EMT, as shown by IPA. Indeed, we have also showed in vitro in both OC cell lines and in *ex-vivo* culture of cells from OC ascites, that EGFR signalling activation increased cell growth with a mechanism involving the cell–cell adhesion protein E-cadherin [29]. Subsequently, others have also identified a transcriptional reprogramming of cell plasticity leading to peritoneal spread mediated by EGFR activation, likely involving a crosstalk with ERBB2 [38]. This latest finding appears to be linked to one of the gene set positively enriched in the MM subgroup, the TRANSMEMBRANE_RECEPTOR_PROTEIN_KINASE_ACTIVITY containing only RTKs which are key cancer molecules linking microenvironment to intra-cellular signalling cascades thus orchestrating in-cell-decision and behaviour. Most of them, when overexpressed or iper-activated due to mutation/s, can cause tumor cell addiction for growth, migration or invasion. Our data are therefore in line with the notion of an increased aggressiveness when more than one of these receptors are co-ordinately up-modulated. RTK activation could be a compensatory pathway to ensure tumor cell aggressiveness [39]. Recently, in ICON7-treated patients, the co-expression of cMET in tumor cells, and VEGFR2 in endothelial cells, has been shown to represent negative prognostic factor only in patients treated with the addition of BEVA [40]. Indeed, the RTK c-MET has been widely studied in tumors resistant to anti-VEGF therapy and associated to worst prognosis in glioblastoma patients treated with BEVA [41, 42].

Our in vitro data suggested that a combination of anti-EGFR drugs with specific RTK inhibitors, i.e. anti-AXL drugs, might have an increased therapeutical effect. Once the relevant cross-talk has been carefully investigated in preclinical models, the employment of combined RTK inhibitors might be therefore considered a promising therapeutic option for those OCs highly expressing EGFR or displaying a primary resistance to EGFR inhibitors. In lung carcinomas efforts are being indeed made to overcome primary or acquired resistance to EGFR inhibitors [35], but these alternative therapeutic approaches still need further preclinical investigations.

Among the molecules/networks predicted by our exploratory IPA analysis, some novel transcriptional connections with EGFR expression and activation emerged. GNA12, encoding for G-alpha subunits of heterotrimeric GTP-binding proteins (G proteins) that link specific cell surface G protein-coupled receptors (GPCRs) to downstream signaling molecules, are intertwined in the largest processes of tumorigenesis and tumor progression [43] and are interesting molecules to be studied in respect to anti-angiogenic therapy. Another interesting emerged gene is NANOG, encoding for a transcription factor regulating the stemness, and likely involved in drug resistance of cancer cells [44]. Of note is AGT, encoding for the angiotensinogen, a component of the renin-angiotensin system (RAS), responsible for the final activation of angiotensin-converting enzymes (ACEs) and that has been shown to be involved in cancer biology and progression [31]. In those individuals suffering with hypertension, the iperactivation of RAS leads to increased hypertension levels as well as to higher incidence of cancer with risk of progression and mortality [45]. In normal ovary, in postmenopausal women, ACE activity was found significantly higher [46] and in an OC xenograft model the inhibition the Angiotensin II, a RAS component, blocked the formation of the tumor spheroids and metastastases in EGFR-dependent manner [47]. Indeed, Angiotensin II promotes OC spheroid formation and metastasis by upregulation of lipid desaturation and suppression of endoplasmic reticulum stress.

## Conclusions

Our data highlight that high and homogeneous cell membrane localization of EGFR, suggestive of receptor activation, can be considered a biomarker of overall survival in OC patients treated with anti-angiogenic agents. This new identified OC subgroup lacking angiogenic molecular characteristics mainly includes histotypes different than high grade serous. Although this group of tumors is very heterogeneous in term of etiology, progression and susceptibility to standard chemotherapy, EGFR expression and EGFR-related signalling activation/s, identified by our bioinformatics analysis, can be considered a commonality. Furthermore, alternative targets could be identified for this particular OC subgroup once the role of EGFR out/in signalling cascade in OC progression will be better clarified. For instance, the possible signalling activated by the crosstalk between EGFR with other RTKs like AXL, as we have shown here, could open the way for multi-target tyrosine kinase inhibitors with important implication in personalized therapeutic strategies.

## Supplementary Information

**Additional file 1: Sup. Table 1.** Patient population of the MITO16A/MaNGO-OV2 in the analysis. **Sup. Table 2.** Association of EGFR membrane expression classified as negative and positive EGFR staining. **Sup. Table 3.** Multivariate analysis of PFS and OS for patients stratified for negative and positive EGFR staning. **Sup. Table 4.** Gene sets analyzed by single-sample GSVA in the MITO16A dataset of gene expression. **Sup. Table 5.** List of genes included in the EGFR-related gene sets. **Sup. Table 6.** Upstream regulators, included in the EGFR-related gene sets, and therir target genes predicted by IPA. **Sup. Table 7.** List of genes included in the networks generated by IPA from EGFR-related gene sets.

**Additional file 2: Supplementary Fig. 1. **Flow diagram of samples entering into the study. NACT, neo-adjuvant chemotherapy; TMA, tissue-macroarray; GE, gene expression. **Supp. Fig. 2.** A. Statistical evaluation of the association of EGFR membrane expression with Ki67 expression. B. Distribution of Ki67 expression in each of the EGFR-generated OC subgroup. **Supp. Fig. 3.A.** Comparison of the patient population analyzed by IHC (left panel) and by gene expression profiling (right panel) stratified for histology. P value, Fisher’s exact text. **B. **Distribution of OC patients according to the molecular signature described previously [26].

## Data Availability

All microarray data were compliant to MIAME (Minimum Information About a Microarray Experiment) guidelines and were deposited into the Gene Expression Omnibus (GEO) database of NCBI (National Center for Biotechnology Expression) (http://www.ncbi.nlm.nih.gov/geo/), with accession numbers GSE208103.

## Abbreviations

OC: Ovarian cancer
PARPi: PARP inhibitors
BEVA: Bevacizumab
RTKs: Receptor tyrosin kinases
HGSOC: High grade serous OC
IHC: Immunohistochemistry
TMAs: Tissue macroarrays
GSEA: Gene set enrichment analysis
IPA: Ingenuity pathway Analysis
GSVA: Gene Set Variation Analysis
PFS: Progression free survival
OS: Overall survival
HRs: Hazard ratios
PS: Performance status
RAS: Renin-angiotensin system
