# Supplementary material for: Biological and clinical impact of membrane EGFR expression in a subgroup of OC patients from the phase IV ovarian cancer MITO-16A/MANGO-OV2A trial
Source: J Exp Clin Cancer Res. 2023 Apr 11;42:83. doi: 10.1186/s13046-023-02651-y (PMC10088260; doi:10.1186/s13046-023-02651-y)
Supplement: Supplementary file 2 — Additional file 2: Supplementary Fig. 1. Flow diagram of samples entering into the study. NACT, neo-adjuvant chemotherapy; TMA, tissue-macroarray; GE, gene expression. Supp. Fig. 2. A. Statistical evaluation of the association of EGFR membrane expression with Ki67 expression. B. Distribution of Ki67 expression in each of the EGFR-generated OC subgroup. Supp. Fig. 3.A. Comparison of the patient population analyzed by IHC (left panel) and by gene expression profiling (right panel) stratified for histology. P value, Fisher’s exact text. B. Distribution of OC patients according to the molecular signature described previously [26]. [file 13046_2023_2651_MOESM2_ESM.pptx]

## Slide 1
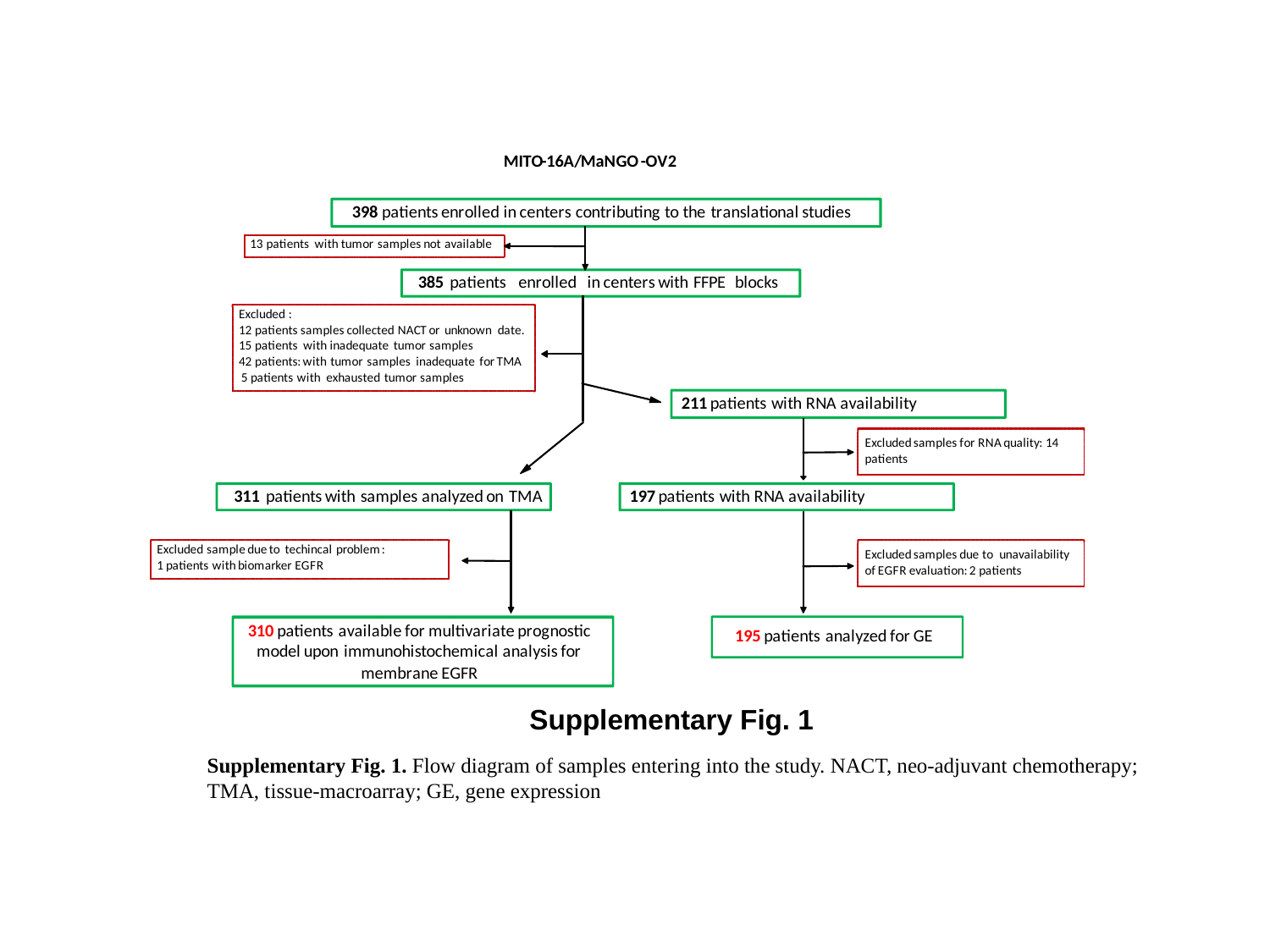

Supplementary Fig. 1
Supplementary Fig. 1. Flow diagram of samples entering into the study. NACT, neo-adjuvant chemotherapy;
TMA, tissue-macroarray; GE, gene expression

## Slide 2
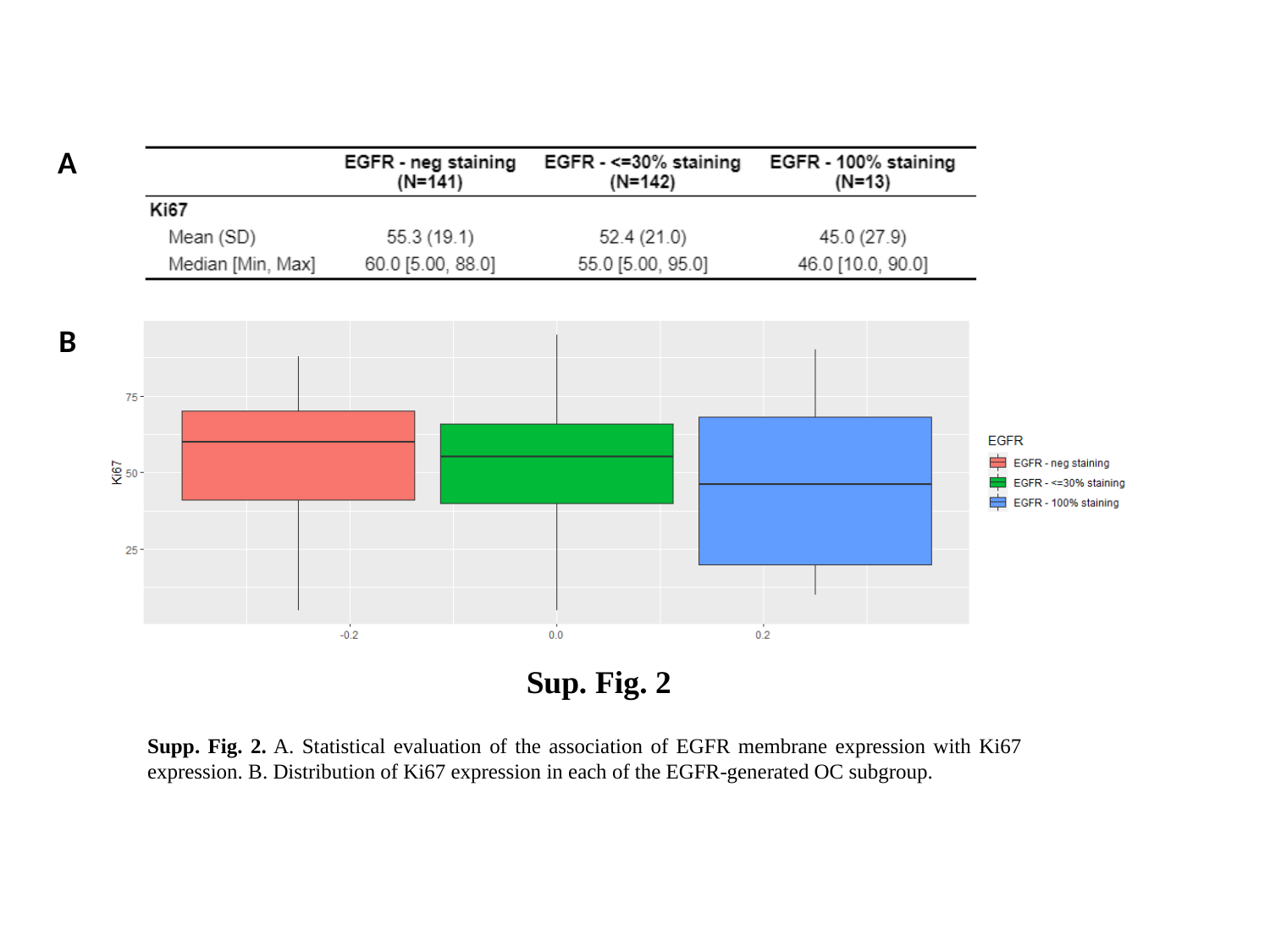

A
B
Sup. Fig. 2
Supp. Fig. 2. A. Statistical evaluation of the association of EGFR membrane expression with Ki67 expression. B. Distribution of Ki67 expression in each of the EGFR-generated OC subgroup.

## Slide 3
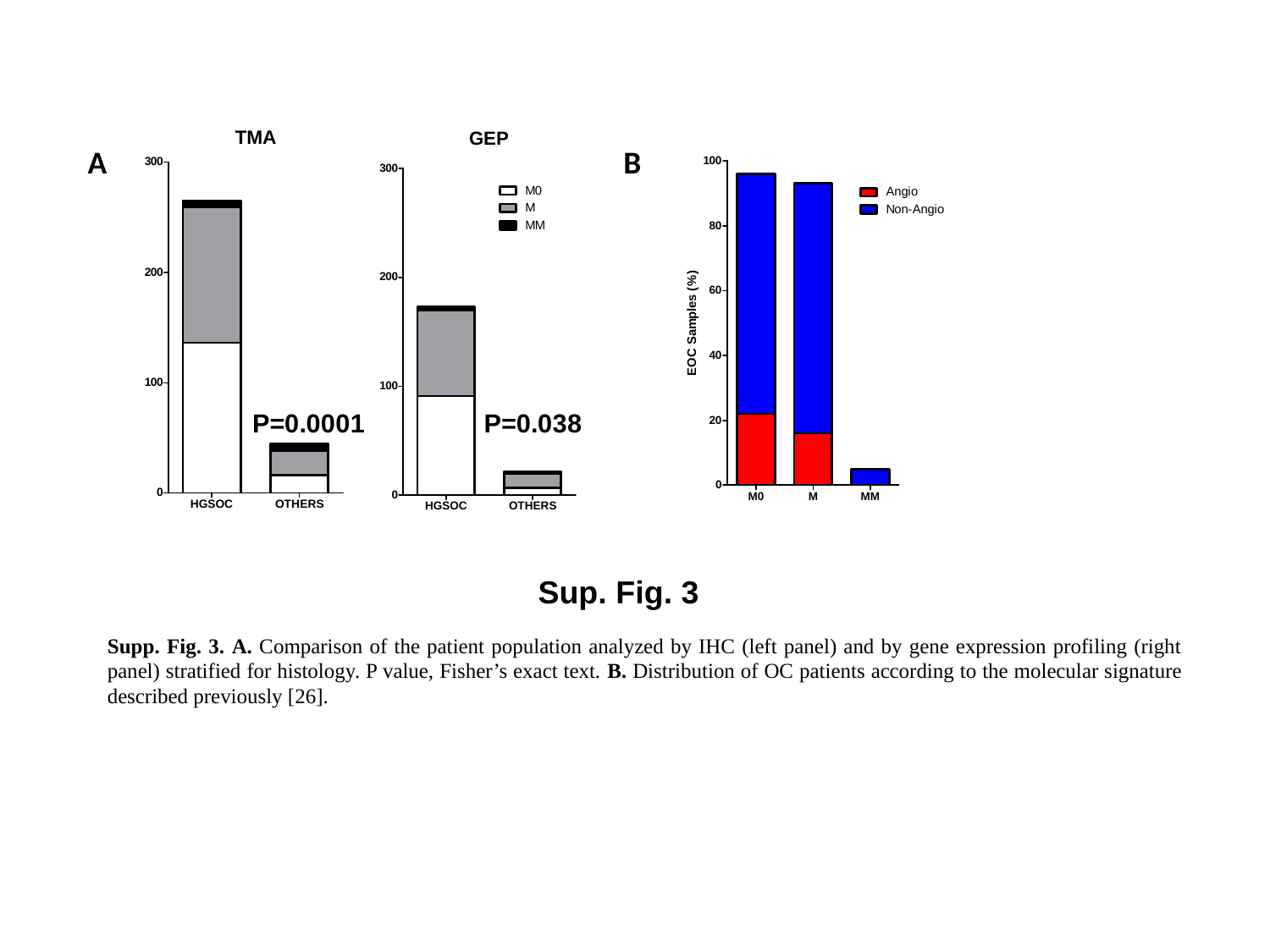

A
B
Sup. Fig. 3
Supp. Fig. 3. A. Comparison of the patient population analyzed by IHC (left panel) and by gene expression profiling (right panel) stratified for histology. P value, Fisher’s exact text. B. Distribution of OC patients according to the molecular signature described previously [26].
